# Admission direct bilirubin and direct bilirubin-to-lymphocyte ratio as exploratory markers of early severity and in-hospital functional status in acute ischemic stroke

**DOI:** 10.3389/fstro.2026.1800285

**Published:** 2026-08-07

**Authors:** Yingxia Li, Ruixing Zhang, Yang Luo, Youquan Gu

**Affiliations:** 1 Department of Neurology, The First Hospital of Lanzhou University, Chengguan District, Lanzhou, Gansu, China; 2 Department of Cardiology, West China Hospital, Sichuan University, Wuhou District, Chengdu, Sichuan, China

**Keywords:** acute ischemic stroke, direct bilirubin, direct bilirubin lymphocyte ratio, early functional outcome, severity

## Abstract

**Objectives:**

To investigate the correlation between direct bilirubin (DBIL), direct bilirubin-lymphocyte ratio (DBLR), and the severity of acute ischemic stroke (AIS), and to evaluate their potential as auxiliary biomarkers for early severity assessment and short-term in-hospital functional outcome of AIS.

**Methods:**

A retrospective study was conducted to enroll 234 patients with AIS who presented within 24 h of admission to The First Hospital of Lanzhou University as the AIS Group, and 180 healthy individuals who underwent a health checkup at the same time were randomly selected as the Control Group. The patients' general information, National Institute of Health Stroke Scale (NIHSS) scores, and laboratory indicators at admission were collected with DBLR calculated as DBIL divided by lymphocyte (LYM) count. The AIS Group was divided into Mild Group (NIHSS ≤ 6, *n* = 91), Moderate Group (6 < NIHSS < 15, *n* = 82), and Severe Group (NIHSS ≥ 15, *n* = 61) according to the NIHSS scores at admission. The AIS Group was further divided into Good early functional outcome Group (mRS < 3, *n* = 133) and Poor early functional outcome Group (mRS ≥ 3, *n* = 101) according to the Modified Rankin Scale (mRS) scores at 7 ± 2 days of treatment. The differences in past medical history, general clinical data, and laboratory indicators between the AIS Group and the Control Group were compared, and the differences in laboratory indicators between the different subgroups were analyzed respectively.

**Results:**

Compared to the Control Group; the AIS Group exhibited significantly higher direct bilirubin (DBIL) levels and a lower lymphocyte (LYM) count (*P* < 0.05). Both the Moderate and Severe Groups presented elevated direct bilirubin (DBIL) levels (*P* < 0.001), indirect bilirubin (IBIL) levels (*P* = 0.021), and direct bilirubin ratio (DBLR) (*P* < 0.001). Notably, DBIL (*r* = 0.269, *P* < 0.001) and DBLR (*r* = 0.321, *P* < 0.001) were positively correlated with the extent of neurological deficit, while LYM count (*r* = −0.274, *P* < 0.001) exhibited a negative correlation with neurological deficit severity. At the time of admission, DBIL and DBLR were identified as risk factors for neurological deficits. This study performed receiver operating characteristic (ROC) curve analysis on DBLR and DBIL among patients with severe strokes. The area under the curve (AUC) for DBLR was 0.612, with an optimal diagnostic threshold of 2.82, yielding a sensitivity of 47.2% and a specificity of 79.4% (*P* = 0.005, 95% CI: 0.537–0.686). For DBIL, the AUC was 0.596, the optimal diagnostic threshold was 2.85, with a sensitivity of 72.7% and a specificity of 43.5% (*P* = 0.017, 95% CI: 0.521–0.671), and the difference was statistically significant (*P* < 0.05). In comparison to the Good early functional outcome Group, the Poor early functional outcome Group showed higher DBIL and DBLR levels, along with a lower LYM count. Moreover, DBIL and DBLR were positively correlated with the Modified Rankin Scale (mRS) scores at 7 ± 2 days. The AUC for DBLR was 0.650, with an optimal diagnostic threshold of 2.86, a sensitivity of 69.8%, and a specificity of 58.5% (*P* < 0.001, 95% CI: 0.581–0.719). The AUC for DBIL was 0.582, with an optimal diagnostic threshold of 2.55, a sensitivity of 80.8%, and a specificity of 36.6% (*P* = 0.031, 95% CI: 0.509–0.654), with a statistically significant difference (*P* < 0.05). Additionally, elevated continuous DBLR was independently associated with poor early functional outcome, and the exploratory cutoff of DBLR > 2.86 indicated a higher risk of poor short-term functional status.

**Conclusions:**

Admission levels of DBIL and DBLR are significantly correlated with the degree of neurological impairment and short-term (7 ± 2 days) in-hospital functional status in AIS patients, where higher values indicate more severe acute neurological deficits and poorer early outcomes. Elevated DBLR is independently associated with poor short-term functional status, and the exploratory cutoff of DBLR > 2.86 (OR = 5.169, 95% CI: 1.623–16.461, *P* = 0.005) may assist in early risk stratification. However, their independent predictive ability is limited (all AUCs < 0.7). Therefore, these biomarkers should only be used as auxiliary tools in combination with clinical scores such as the NIHSS, and their clinical value should not be overinterpreted.

## 1 Introduction

Globally, stroke is still the disease with the highest morbidity and mortality, of which about 80%−85% is ischemic stroke (Barthels and Das, 2020; Khan et al., 2024; Wang et al., 2017). Although timely intravenous thrombolytic therapy or endovascular interventional therapy can improve the clinical outcomes of acute ischemic stroke (AIS) patients to a certain extent, only less than 50% of patients can achieve timely recanalization, and some patients still have poor prognosis after timely recanalization (von Kummer et al., 2015). Bilirubin has been considered as a kind of metabolic waste, and more and more studies (Sheng et al., 2021) have proved that it has an anti-inflammatory effect and can also affect the expression of factors related to inflammatory mechanisms in some diseases. In addition, studies have found that bilirubin levels can predict the occurrence of certain vascular events, such as the total mortality of coronary heart disease, peripheral vascular disease, diabetic amputation patients, and diabetic nephropathy (Liu et al., 2017).

In clinical practice, bilirubin is reported as direct bilirubin (DBIL) and total bilirubin (TBIL), with TBIL being the sum of DBIL and indirect bilirubin (IBIL). A previous study (Zhong et al., 2020) showed that bilirubin had neuroprotective effects. However, there is still no consensus on whether bilirubin affects the prognosis of AIS. Meanwhile, although most of previous studies (Sheng et al., 2021; Ouyang et al., 2021) have analyzed the role of serum TBIL on AIS patients, they have not further subdivided TBIL into different types and analyzed how they affected the severity and prognosis of AIS. There is still a lack of information about the role of combination of DBIL and lymphocyte (LYM) ratio on the severity and prognosis of AIS patients. It is worth noting that, most of these studies (Zhong et al., 2020) have focused on the relationship between bilirubin levels and the prevention of ischemic stroke, with little discussion on changes in bilirubin levels when stroke occurs.

Therefore, this study will further explore the relationship between the bilirubin level at admission and the severity and early functional outcome of AIS patients, and analyze the relationship between the DBIL, IBIL, TBIL, direct bilirubin lymphocyte ratio (DBLR) and AIS in detail.

## 2 Study cohort

### 2.1 Subjects

A total of 234 patients with acute ischemic stroke (AIS) who were admitted to The First Hospital of Lanzhou University within 24 h of symptom onset were enrolled in the AIS Group. Additionally, 180 healthy individuals who underwent health checkups during the same period were randomly selected to serve as the Control Group.

The AIS Group was divided according to the National Institute of Health Stroke Scale (NIHSS) (Kwah and Diong, 2014) scores at admission. There were 91 patients in the Mild Group (NIHSS ≤ 6), 82 patients in the Moderate Group (6 < NIHSS < 15), and 61 patients in the Severe Group (NIHSS ≥ 15). According to the Modified Rankin Scale (mRS) (Haggag and Hodgson, 2022) scores at 7 ± 2 days, the AIS Group was divided into Good early functional outcome Group (mRS < 3) and Poor early functional outcome Group (mRS ≥ 3).

This study was approved by the Ethics Committee of The First Hospital of Lanzhou University (NO. LDYYLL-2024-737).

### 2.2 Inclusion criteria

(1) Age ≥ 18 years old; (2) Ischemic stroke confirmed by admission signs and imaging examinations (cranial CT/MRI); (3) Definite symptoms or signs of neurological impairment; (4) Admission within 24 hours after symptom onset; (5) mRS ≤ 2 before onset; (6) Complete clinical data available and informed consent obtained from patients' families.

### 2.3 Exclusion criteria

**Figure 1:**
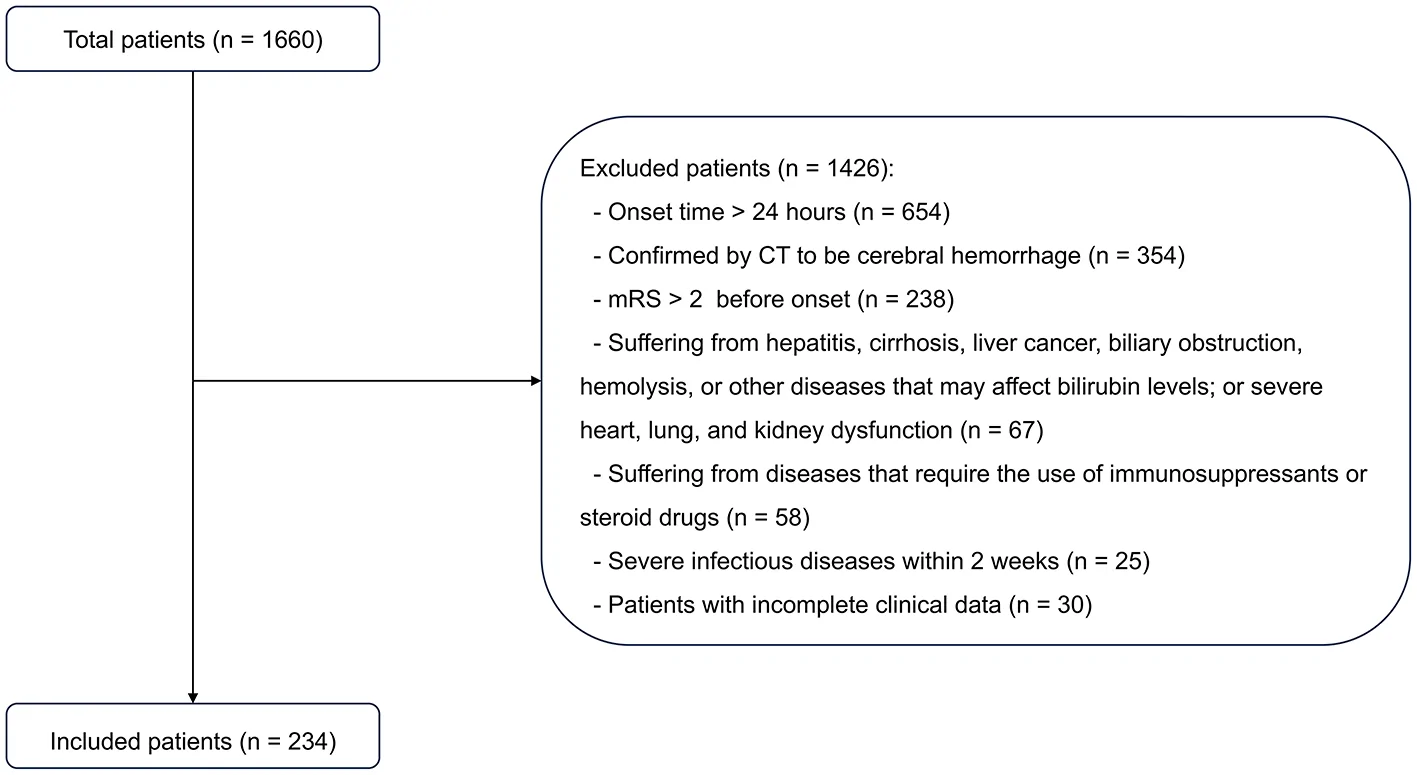
The process of how to select subjects in this study.

(1) Time from onset to admission > 24 h; (2) Cerebral hemorrhage confirmed by CT with similar symptoms; (3) mRS > 2 before onset; (4) Severe infectious diseases within 2 weeks; (5) Severe diseases requiring immunosuppressive agents or steroids; (6) Patients with diseases that may affect bilirubin levels (hepatitis confirmed by serum hepatitis virus markers, cirrhosis confirmed by imaging and liver function tests, liver cancer confirmed by pathology or imaging, biliary obstruction confirmed by imaging, hemolysis confirmed by blood routine and reticulocyte count) or severe heart, lung, and kidney dysfunction (NYHA cardiac function grade ≥ III, respiratory failure requiring mechanical ventilation, renal insufficiency with eGFR < 30 mL/min/1.73m^2^); (7) Incomplete clinical data. The process of how to select subjects in this study is presented in Figure 1.

## 3 Methods

### 3.1 General information collection

Trained professionals collected baseline demographic characteristics, vascular risk factors (hypertension, diabetes, atrial fibrillation, and coronary heart disease), height, weight, body mass index (BMI), and NIHSS scores. The inter-rater reliability of NIHSS and mRS scoring was verified, with Kappa values of 0.87 and 0.85 respectively, indicating good consistency.

### 3.2 Laboratory examination

Cubital venous blood samples were collected from all subjects upon admission, and lymphocyte (LYM) counts were determined using an automatic cell analyzer. Fasting elbow venous blood samples were obtained within 24 h of admission to assess TBIL, DBIL, and IBIL through biochemical analysis. The DBLR was calculated by dividing DBIL (μmol/L) by the LYM count ( × 10^9^/L).

### 3.3 Assessment of the severity of the disease

The severity of stroke at the time of onset was evaluated using the NIHSS scores, which comprehensively assess patients' consciousness, limb muscle strength, speech function, and other neurological impairments, with scores ranging from 0 to 42. Based on this scale, the severity of stroke is classified as mild, moderate, or severe. Patients underwent further assessment 7 ± 2 days post-stroke to evaluate short-term neurological recovery using the mRS scores. Generally, patients with an mRS score of ≥ 3 are considered to have relatively poor neurological recovery and varying degrees of disability. All participating clinicians were certified neurologists with professional training.

### 3.4 Statistical analysis

SPSS 27.0 software was used for statistical analysis. Measurement data with normal distribution were expressed as *x* ± *s*, and differences between groups were analyzed by *t*-test. Measurement data that did not meet normal distribution or homogeneity of variance (laboratory indicators, time from onset to admission, NIHSS scores) were described as median (Q1, Q3). Kruskal-Wallis rank sum test was used for comparison among multiple groups, and Dunn-Bonferroni was used for *post-hoc* test. Mann–Whitney U test was used for comparison between two independent samples. Count data were described by frequency and percentage, and chi-square test or Fisher exact test was used for comparison between groups. Spearman rank correlation was used to analyze the correlation between stroke severity, early functional outcome, and laboratory indicators.

Significant variables in univariate analysis were included in the model (stepwise regression method). Separate ordinal Logistic regression models were constructed for DBIL, lymphocyte count, and DBLR respectively to avoid statistical bias from mathematically interdependent variables, and used to evaluate independent risk factors for stroke severity. Binary Logistic regression model was used to assess independent predictors of poor early functional outcome, with odds ratio (OR) and 95% confidence interval (CI) calculated. The proportional odds assumption for ordinal logistic regression was verified by the Brant test.

ROC curve was used to evaluate the predictive value of DBIL and DBLR levels for AIS severity and early functional outcome, and the combined predictive value of DBIL and DBLR was also analyzed. Two-sided *P* < 0.05 was considered statistically significant.

**Figure 2:**
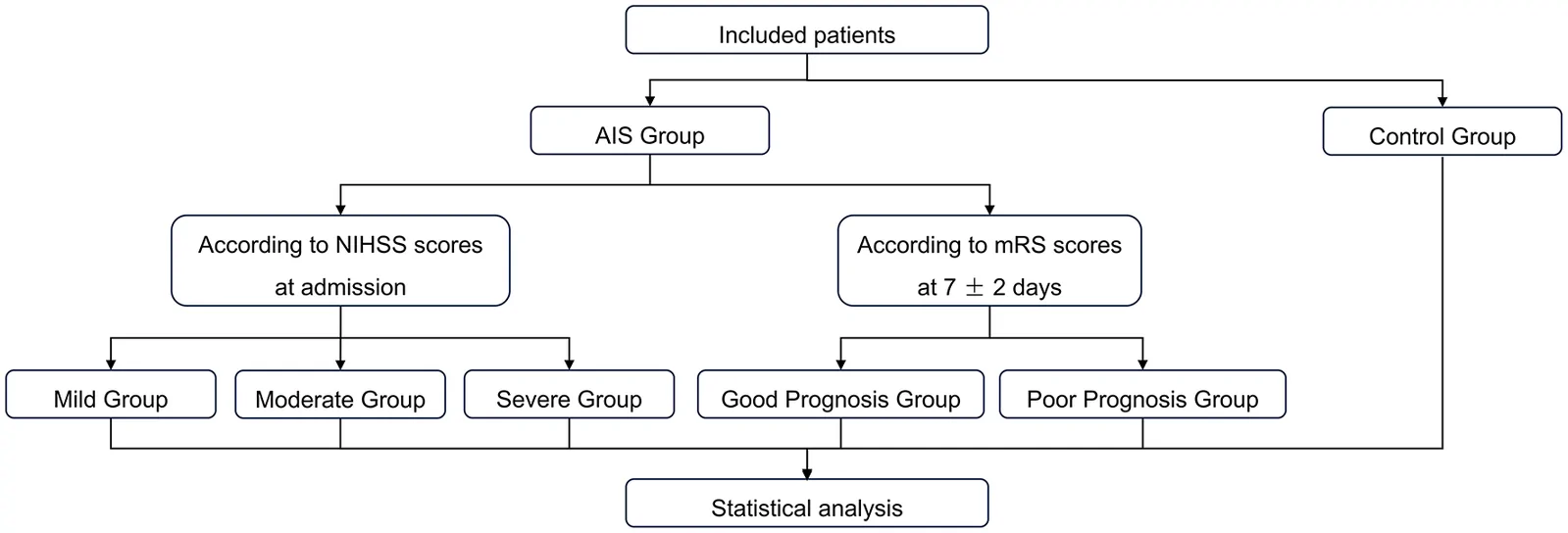
The overall process of this study (AIS, acute ischemic stroke; NIHSS, National Institute of Health Stroke Scale; mRS, Modified Rankin Scale).

The overall process of this study is shown in Figure 2.

## 4 Results

### 4.1 Comparison of baseline characteristics between the AIS group and control group

A total of 234 patients with AIS were included in this study, comprising 151 males (64.5%) and 83 females (35.5%). The ages of the participants ranged from 24 to 94 years, with a mean age of 68.54 ± 12.5 years. The control group consisted of 180 healthy individuals, including 100 males (55.6%) and 80 females (44.4%), with a mean age of 68.47 ± 8.54 years. Compared to the control group, the AIS group exhibited a significantly higher incidence of hypertension (66.2% vs. 51.7%, χ^2^ = 12.236, *P* < 0.001). Additionally, the AIS group showed higher rates of diabetes (28.2% vs. 24.4%), atrial fibrillation (32.9% vs. 25.0%), and coronary heart disease (16.7% vs. 16.1%), as well as higher systolic blood pressure (143.3 ± 22.48 vs. 141.80 ± 25.78 mmHg), diastolic blood pressure (82.62 ± 15.48 vs. 79.28 ± 15.30 mmHg), and body mass index (BMI) (23.81 ± 3.45 vs. 22.89 ± 3.57 kg/m^2^). However, these differences were not statistically significant *(P* > 0.05*)*. The comparison of demographic characteristics and cerebrovascular risk factors between the two groups is presented in Table 1.

**Table 1:** Comparison of baseline characteristics between the AIS group and control group.

	AIS Group (*n* = 234)	Control group (*n* = 180)	*X^2^/F* value	*P* value
Sex			2.841^**a**^	0.092
Male	151 (64.5%)	100 (55.6%)		
Female	83 (35.5%)	80 (44.4%)		
Age (years)	68.54 ± 12.5	68.47 ± 8.54	0.004^**b**^	0.947
Previous history (*n*[%])				
Hypertension	155 (66.2%)	87 (51.7%)	12.23^**a**^	**< 0.001**
Diabetes	66 (28.2%)	44 (24.4%)	0.159^**a**^	0.690
Atrial fibrillation	77 (32.9%)	45 (25.0%)	2.773^**a**^	0.096
Coronary heart disease	39 (16.6%)	29 (16.1%)	0.009^**a**^	0.925
Personal history (*n*[%])				
Smoking	38 (16.2%)	40 (22.2%)	2.576^**a**^	0.108
Drinking	24 (10.2%)	27 (15.0%)	2.663^**a**^	0.132
Systolic blood pressure (mmHg)	143.3 ± 22.48	141.80 ± 25.78	1.539^**b**^	0.652
Diastolic blood pressure (mmHg)	82.62 ± 15.48	79.28 ± 15.30	0.048^**b**^	0.128
BMI (kg/m^2^)	23.81 ± 3.45	22.89 ± 3.57	0.034^**b**^	0.065

^a^is *X*^2^ value;

^b^is *F* value.

### 4.2 Comparison of laboratory indicators between the AIS group and control group

Compared with the Control Group, the AIS Group had higher DBIL (median [3.50 vs. 2.70] μmol/L, *Z* = 23.096, *P* < 0.001), higher TBIL (median [16.20 vs. 15.40] μmol/L, *Z* = 1.194, *P* = 0.233, no statistical significance), and lower LYM count (median [1.22 vs. 1.41] × 109/L, *Z* = 5.770, *P* = 0.016). There was no significant difference in IBIL between the two groups (*P* = 0.593; Table 2).

**Table 2:** Comparison of laboratory indicators between the AIS group and control group.

	AIS group (*n* = 234)	Control Group (*n* = 180)	*Z* value	*P* value
LYM count ( × 109/L) M(Q1, Q3)	1.22 (0.78, 1.69)	1.41 (0.11, 6.31)	5.770	0.016
TBIL (μmol/L) M(Q1, Q3)	16.20 (12.23, 22.38)	15.40(5.80, 36.20)	1.194	0.233
DBIL (μmol/L) M(Q1, Q3)	3.50 (2.50, 4.90)	2.70(0.90, 9.70)	23.096	**< 0.001**
IBIL (μmol/L) M(Q1, Q3)	12.60 (9.30, 17.50)	12.50(2.50, 29.40)	0.189	0.593

### 4.3 Distribution of meaningful experimental measures between subgroups with different NIHSS scores

**Figure 3:**
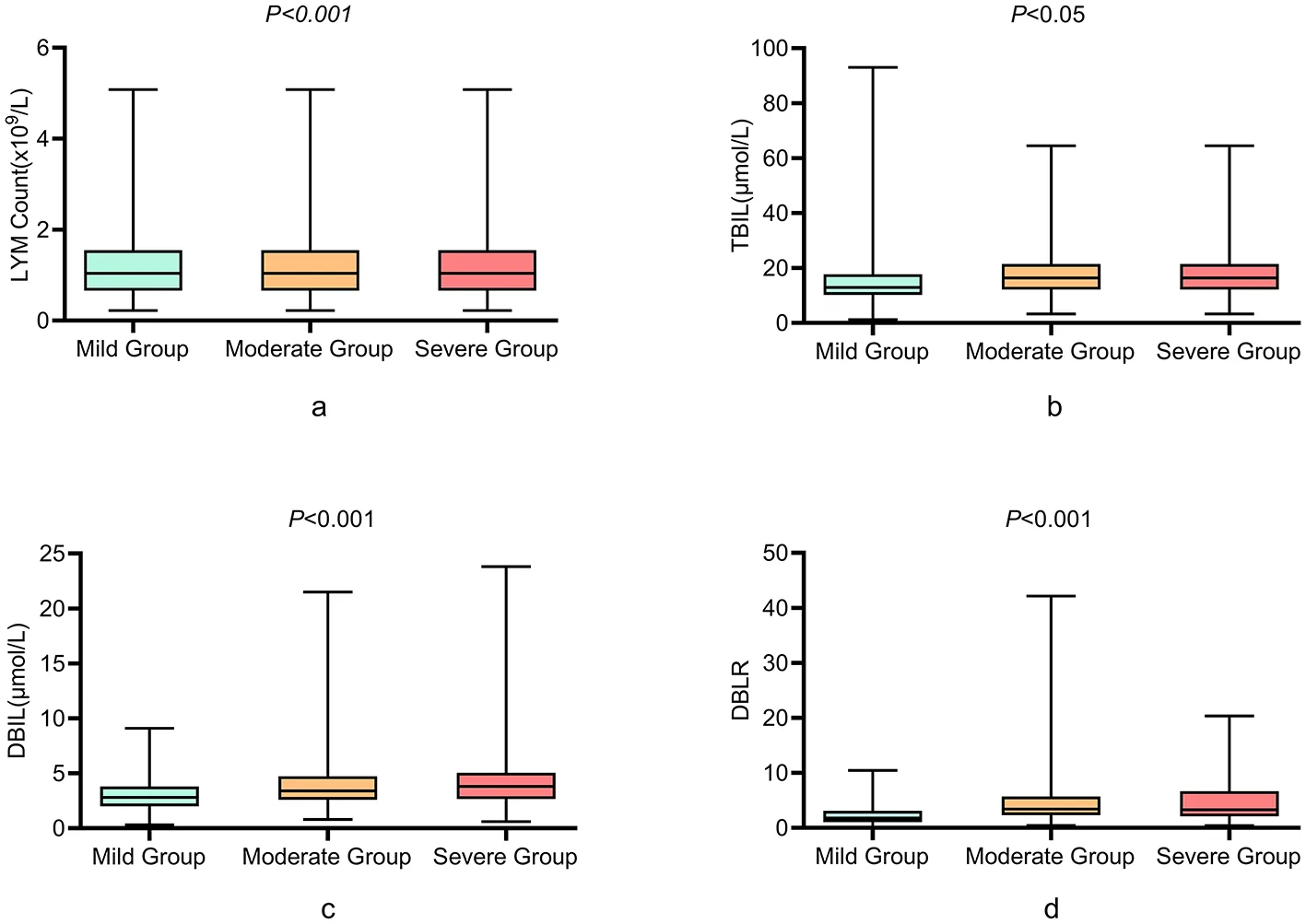
Distribution of laboratory indicators among subgroups with different NIHSS scores. **(a)**: LYM count; **(b)**: TBIL; **(c)**: DBIL; **(d)**: DBLR. *P* < 0.05 vs. mild group; *P* < 0.05 vs. moderate group.

Compared with Mild Group, Moderate Group, and Severe Group had higher TBIL (median [12.30 vs. 16.40 vs. 15.10] μmol/L, *Z* = 8.199, *P* = 0.017), DBIL (median [2.85 vs. 4.10 vs. 4.70] μmol/L, *Z* = 18.917, *P* < 0.001), IBIL (median [9.60 vs. 11.02 vs. 13.70] μmol/L, *Z* = 7.711, *P* = 0.021), DBLR(median [1.99 vs. 3.61 vs. 3.75], *Z* = 29.989, *P* < 0.001), and lower LYM count (1.50 [1.00, 1.96] vs. 1.01 [0.71, 1.42] vs. 1.00 [0.66, 1.54] × 109/L, *Z* = 21.780, *P* < 0.001), with statistically significant differences among the three groups (*P* < 0.05) (Table 3 and Figure 3).

**Table 3:** Distribution of meaningful experimental measures between subgroups with different NIHSS scores.

	Mild group (*n* = 91)	Moderate group (*n* = 82)	Severe group (*n* = 61)	*Z* value	*P* value
LYM count ( × 109/L) M (Q1, Q3)	1.50 (1.00, 1.96)	1.01 (0.71, 1.42)^**a**^	1.00 (0.66, 1.54)^b^	21.780	**< 0.001**
TBIL (μmol/L) M(Q1, Q3)	12.30 (11.00, 24.20)	16.40 (11.00, 19.03)^**a**^	15.10 (12.70, 22.60)^**b**^	8.199	**0.017**
DBIL (μmol/L) M(Q1, Q3)	2.85 (2.43, 4.33)	4.10 (3.20, 6.50)^**a**^	4.70 (3.40, 5.30)^**b**^	18.917	**< 0.001**
IBIL (μmol/L) M(Q1, Q3)	9.60 (8.20, 19.40)	11.02(8.90, 14.05)	13.10 (9.70, 14.80)^**b**^	7.711	**0.021**
DBLR	1.99 (1.19, 3.22)	3.61 (2.32, 6.22)^a^	3.75 (2.19, 6.87) ^b^	29.989	**< 0.001**

^a^Compared with Mild Group, *P* < 0.05.

^b^Compared with Moderate Group, *P* < 0.05.

### 4.4 The analysis of the correlation between laboratory indicators and NIHSS scores in the AIS Group

Spearman correlation analysis showed that DBIL (*r* = 0.269*, P* < 0.001) and DBLR (*r* = 0.321, *P* < 0.001) were positively correlated with NIHSS scores at admission, while LYM count (*r* = –0.274, *P* < 0.001) was negatively correlated with NIHSS scores (Table 4).

**Table 4:** Correlation between laboratory indicators and NIHSS score in the AIS group.

	LYM count	DBIL	DBLR
*r*	**−0.274**	0.269	0.321
*P* value	< 0.001	< 0.001	< 0.001

### 4.5 The analysis of risk factors for the degree of neurological deficit in the AIS group

Multivariate ordinal Logistic regression analysis (stepwise regression) showed that DBIL (OR = 1.581, 95%CI:1.223–2.043, *P* < 0.001) and DBLR (OR = 0.815, 95%CI:0.683–0.972, *P* = 0.023) were risk factors for the degree of neurological deficit in AIS patients (Table 5).

**Table 5:** Multivariate ordinal Logistic regression analysis of the degree of neurological deficit in the AIS group.

	*B* value	*SE* value	Wald value	*P* value	OR value	95%CI value
Atrial fibrillation	−0.085	0.0306	0.078	0.780	0.920	0.378–3.575
LYM count	0.142	0.2296	0.384	0.535	1.153	0.735–1.809
TBIL	−0.030	0.0398	0.564	0.453	0.971	0.898–1.049
DBIL	0.458	0.1309	12.241	**< 0.001**	**1.581**	1.223–2.043
IBIL	0.033	0.0407	0.644	0.422	1.033	0.954–1.119
DBLR	−0.205	0.0900	5.173	**0.023**	**0.815**	0.683–0.972

To avoid statistical bias caused by mathematically interdependent variables (DBIL, LYM, and DBLR; TBIL, DBIL, and IBIL), we constructed three separate ordinal logistic regression models, adjusted for age, sex, hypertension, diabetes, atrial fibrillation, and coronary heart disease.

As shown in Table S1, elevated DBIL (OR = 1.581, 95%CI: 1.223–2.043, *P* < 0.001), decreased lymphocyte count (OR = 0.724, 95%CI: 0.602–0.871*, P* = 0.001), and elevated DBLR (OR = 1.326, 95%CI: 1.087–1.618, *P* = 0.006) were all independently associated with increased stroke severity. All models satisfied the proportional odds assumption (Brant test, all *P* > 0.05).

### 4.6 ROC curves of DBLR and DBIL at admission predicting severe stroke

**Figure 4:**
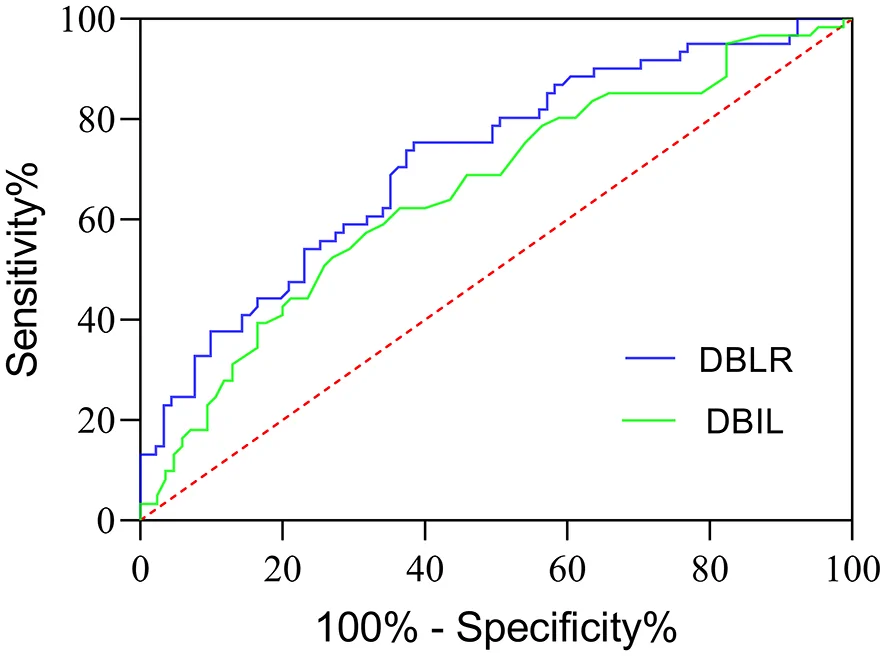
ROC curves of DBLR and DBIL at admission predicting severe stroke.

ROC curve analysis was used to analyze the DBLR and DBIL at admission predicting severe stroke. The results showed that the AUC of DBLR was 0.612, the best diagnostic value was 2.82, the sensitivity was 47.2%, and the specificity was 79.4% (*P* = 0.005, 95%CI: 0.537–0.686). The AUC of DBIL was 0.596, the best diagnostic value was 2.85, the sensitivity was 72.7%, and the specificity was 43.5% (*P* = 0.017, 95%CI: 0.521–0.671), the difference was statistically significant (*P* < 0.05) (Table 6 and Figure 4).

**Table 6:** ROC curves of DBLR and DBIL at admission predicting severe stroke.

	Best diagnostic value	Sensitivity (%)	Specificity (%)	AUC	SE value	*P* value	95%CI
DBLR	2.82	47.2	79.4	**0.612**	0.038	0.005	0.537–0.686
DBIL	2.85	72.7	43.5	**0.596**	0.038	0.017	0.521–0.671

### 4.7 Comparison of laboratory indicators between good early functional outcome group and poor early functional outcome group

**Figure 5:**
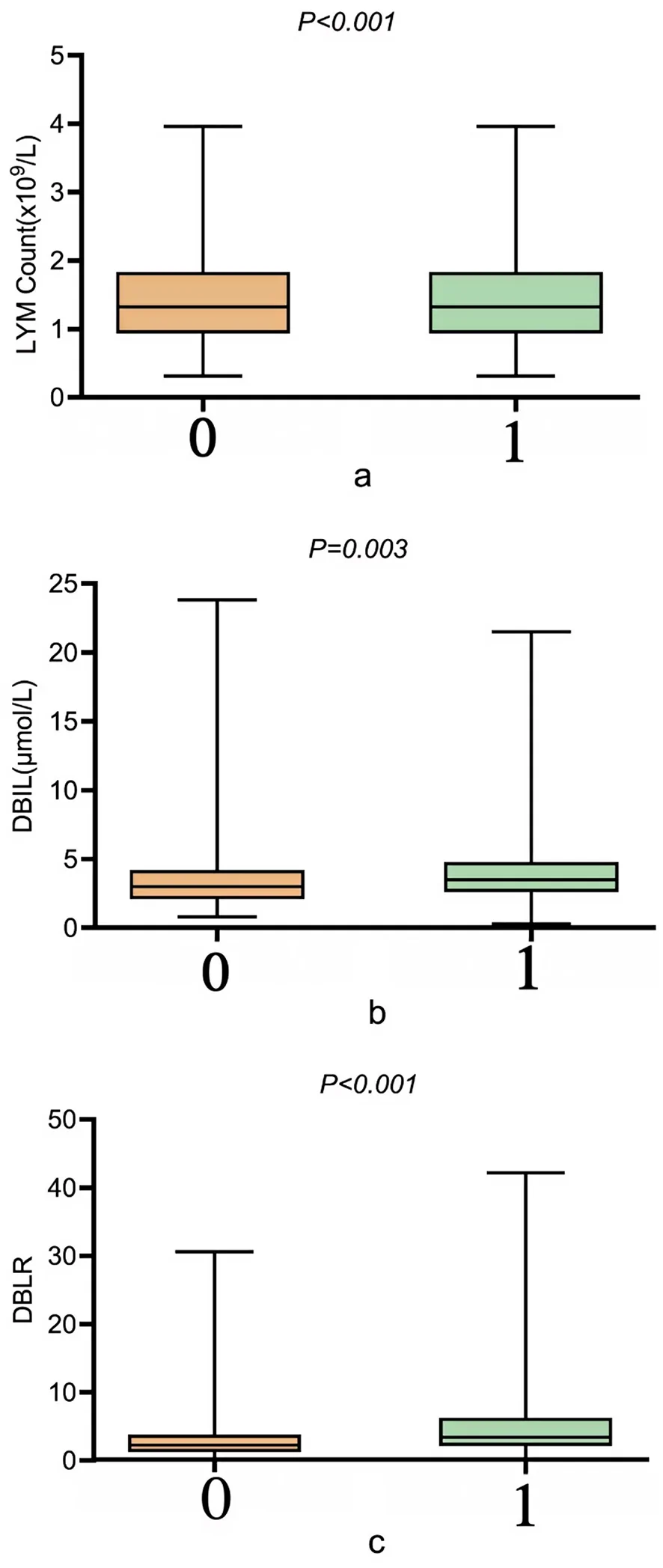
Distribution map of meaningful indicators between Good early functional outcome Group and Poor early functional outcome Group. **(a)** LYM count; **(b)** DBIL; **(c)** DBLR. (0 = Good early functional outcome Group; 1 = Poor early functional outcome Group).

Compared with the Good early functional outcome Group, the Poor early functional outcome Group had higher DBIL (median [3.05 vs. 4.70] μmol/L, *Z* = −2.854, *P* = 0.004) and DBLR (median (2.72 vs. 4.24), *Z* = −4.004, *P* < 0.001). There was a lower median LYM count ([1.10 vs. 0.74] × 109/L, *Z* = −3.783, *P* < 0.001), and the difference between the two groups was statistically significant (*P* < 0.05) (Table 7 and Figure 5).

**Table 7:** Comparison of laboratory indicators between good early functional outcome group and poor early functional outcome group.

	Good early functional outcome group (*n* = 133)	Poor early functional outcome group (*n* = 101)	*Z* value	*P* value
LYM count ( × 109/L) M(Q1,Q3)	1.10 (0.63, 1.46)	0.74 (0.64, 1.24)	−3.783	**< 0.001**
TBIL (μmol/L) M(Q1,Q3)	14.80 (12.2, 19.2)	16.90 (10.95, 22.52)	−1.394	0.163
DBIL (μmol/L) M(Q1,Q3)	3.05 (2.75, 4.52)	4.70 (3.28, 5.30)	−2.854	**0.004**
IBIL (μmol/L) M(Q1,Q3)	11.32 (9.00, 15.10)	13.70 (8.73, 17.78)	−1.617	0.106
DBLR	2.72 (0.90, 8.93)	4.24 (3.35, 8.64)	−4.004	**< 0.001**

### 4.8 Correlation between indices and mRS scores at 7±2 days in AIS patients

Spearman correlation analysis showed that NIHSS scores (*r* = 0.516, *P* < 0.001), DBIL (*r* = 0.152, *P* = 0.027), and DBLR (*r* = 0.220, *P* = 0.001) at admission were positively correlated with the mRS scores at 7 ± 2 days. LYM count (*r* = −0.205, *P* = 0.002) was negatively correlated with 7 ± 2 days mRS scores (Table 8).

**Table 8:** Correlation between indices and mRS scores at 7 ± 2 days in AIS patients.

	NIHSS scores at admission	LYM	DBIL	DBLR
*r*	0.516	–**0.205**	0.152	0.220
*P* Value	< 0.001	0.002	0.027	0.001

### 4.9 ROC curves of DBLR and DBIL at admission predicting poor early functional outcome in AIS patients

**Figure 6:**
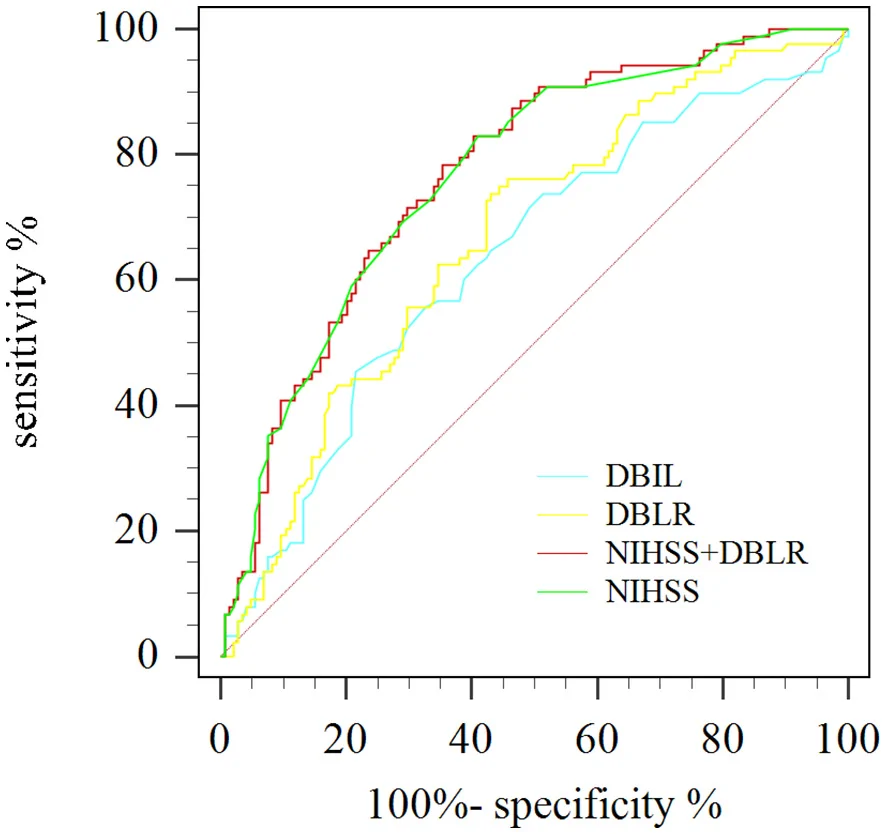
ROC curves of DBIL, DBLR, NIHSS and combined NIHSS+DBLR model at admission predicting poor early functional outcome in AIS patients.

In this study, the DBLR and DBIL at the admission of the Good early functional outcome Group and Poor early functional outcome Group were analyzed by ROC curves. The results showed that the AUC of DBLR was 0.650, the best diagnostic value was 2.86, the sensitivity was 69.8%, and the specificity was 58.5% (*P* < 0.001, 95%CI: 0.581–0.719). The AUC of DBIL was 0.582, the best diagnostic value was 2.55, the sensitivity was 80.8%, and the specificity was 36.6% (*P* = 0.031, 95%CI: 0.509–0.654). The difference was statistically significant (*P* < 0.05) (Table 9 and Figure 6).

**Table 9:** ROC curves of DBLR, DBIL, NIHSS and combined model for predicting poor early functional outcome in AIS patients.

	Best diagnostic value	Sensitivity (%)	Specificity (%)	AUC	SE	*P* value	95%CI
DBLR	2.86	69.8	58.5	**0.650**	0.035	0.000	0.581–0.719
DBIL	2.55	80.8	36.6	**0.582**	0.037	0.031	0.509–0.654
NIHSS	8	83	59.1	**0.767**	0.031	0.000	0.706–0.820
NIHSS+DBLR		78.4	65.1	**0.769**	0.038	0.000	0.708–0.830

Pairwise comparisons of ROC curves using DeLong's test are summarized in Table S2. DBLR alone showed significantly lower AUC compared with NIHSS alone (ΔAUC = 0.101, *P* = 0.020) and compared with the combined NIHSS+DBLR model (ΔAUC = 0.103, *P* = 0.014). However, adding DBLR to NIHSS did not significantly improve the AUC (ΔAUC = 0.002, *P* = 0.782), suggesting that the prognostic information captured by DBLR is largely overlapping with that of NIHSS. No significant difference was observed between DBLR and DBIL (ΔAUC = 0.033, *P* = 0.529).

### 4.10 Analysis of risk factors for poor early functional outcome

Whether the NIHSS scores, LYM count, DBIL, and DBLR at admission was greater than the critical value in univariate analysis was used as independent variables, and the DBLR value greater than the critical value was assigned a value of 1, and less than the critical value was 0. The prognostic outcome was considered as the dependent variable. Multivariate binary Logistic regression analysis was performed. Logistic regression analysis showed that NIHSS scores at admission (OR = 1.127, *P* < 0.001) and continuous DBLR (OR = 1.234, *P* = 0.001) were independently associated with poor early functional outcome (Table 10).

**Table 10:** Analysis of risk factors for poor early functional outcome of AIS patients.

	*B* value	*SE* value	*Wald* value	*P* value	*OR* value	95%CI
NIHSS at admission	0.119	0.028	17.738	**< 0.001**	1.127	1.066–1.191
LYM	0.286	0.382	0.562	0.454	1.331	0.630–2.813
DBIL	0.087	0.094	0.848	0.357	1.091	0.886–1.262
DBLR > 2.86	1.643	0.591	7.725	**0.005**	5.169	1.623–16.461

## 5 Discussion

AIS poses a severe threat to human health due to its limited and strict time-dependent treatment. At current medical levels, the disability and mortality rates are as high as 50%. Therefore, finding simple and accurate serum biomarkers to evaluate the degree of early neurological damage and early functional outcome in stroke patients is shown to be exploratory association. This retrospective study assesses the value of DBIL and DBLR in predicting early neurological deficits and poor early functional outcome, confirming the relationship between DBIL and the severity and early functional outcome of AIS through subgroup analysis, and suggesting that DBLR may be an effective indicator for predicting stroke severity and early functional outcome. DBIL and DBLR are low-cost blood biomarkers with easily obtainable standardized blood samples in emergency settings. This study finds that DBIL and DBLR are correlated with the degree of early neurological dysfunction and poor early functional outcome in patients, but their predictive value is relatively weak (AUC < 0.7), and DBLR has better predictive ability than DBIL. In addition, the optimal cut-off values of DBIL and DBLR for predicting severity and poor early functional outcome are determined.

Recent large-cohort studies have further elucidated the relationship between bilirubin subtypes and outcomes in stroke patients. Xu et al. (2026) analyzed 787 AIS patients undergoing mechanical thrombectomy and found that elevated DBIL and TBIL were positively associated with stroke-associated pneumonia, intracranial hemorrhage, and 3-month poor functional outcome, with DBIL showing superior predictive value over other bilirubin subtypes (Xu et al., 2026). Similarly, Qiu et al. (2025). demonstrated in 914 patients with intracerebral hemorrhage that higher serum TBIL levels were independently associated with increased 28-day mortality (HR = 1.121, 95% CI:1.063–1.182) (Qiu et al., 2025). These findings, together with our results, support the notion that bilirubin—particularly DBIL—may serve as a useful biomarker for early functional outcome in acute cerebrovascular events, although the underlying mechanisms require further investigation.

### 5.1 Correlation between bilirubin and AIS

Bilirubin is the end product of heme metabolism and becomes really harmful when it accumulates in too high concentrations in tissues (Rovira et al., 2005). However, high serum bilirubin levels seem to have a protective effect against diseases caused by oxidative stress. However, little is known about these changes and their role in the acute phase of ischemic stroke. Some studies (Yu et al., 2021) showed that the risk of stroke decreased with increasing bilirubin levels. However, this association was not evident for either hemorrhagic stroke or female patients. Another study (Sun et al., 2024) concluded that stroke patients had relatively high levels of all types of bilirubin, and male patients had significantly higher levels of all types of bilirubin than female patients (*P* < 0.05). Elevated serum levels of DBLR and TBIL had also been introduced as indicators of stroke severity after the onset of ischemic stroke. In a study by Arsalan et al. (2011), high serum bilirubin levels predicted the severity of stroke and prolonged the duration of treatment in the acute phase of stroke. A later study (Ziberna et al., 2016) found that the protective effect of bilirubin on ischemic stroke was related to its antioxidant properties. Experimental data (Thakkar et al., 2019) showed that bilirubin could play an endogenous antioxidant effect by inhibiting the oxidation of low-density lipoprotein and cholesterol, and prevent and inhibit atherosclerosis by inhibiting the migration and proliferation of monocytes and vascular smooth muscle cell inflammation and vascular endothelial dysfunction. It was further found that bilirubin level was significantly negatively correlated with carotid atherosclerotic plaque. Secondly, in addition to inhibiting vascular atherosclerosis to prevent stroke, bilirubin could also prevent stroke by inhibiting thrombosis. Bilirubin could also inhibit neuronal damage after stroke. The underlying mechanisms were as follows: (1) Heme oxygenase (HO), an enzyme of bilirubin, exerts neuroprotective effects. An experimental study (Arsalan et al., 2011) showed that overexpression of HO could limit neuronal damage, and when HO was knocked out, the nerve damage was aggravated. However, the neurological damage after this knockout could be reversed by restoring low concentrations of bilirubin. (2) Bilirubin provides far more neuroprotection than glutathione. Because of the rapid cycling of biliverdin reductase, bilirubin can protect cells from excessive oxidant damage by 10,000 times. The increased expression of biliverdin reductase in experimental studies (Qaisiya et al., 2014) further supported the neuroprotective effect of bilirubin. These data indirectly supported the protective role of bilirubin in neuronal injury. Li et al. (2014) included 13,000 healthy United States adults and found that high levels of bilirubin were inversely associated with the prevalence of stroke. People with the highest bilirubin levels had a 44% reduction in the risk of stroke. In addition, a higher bilirubin level was found to be associated with lower disability and higher capacity for neurological rehabilitation in patients with previous stroke, suggesting that a higher bilirubin level was associated with better recovery in patients with stroke. These findings supported the hypothesis that bilirubin might be an important endogenous defense mechanism in stroke events and might protect the nerve system from injury when stroke occurs. In addition to the above effects, bilirubin was also found to have complex immunomodulatory effects (Li et al., 2020). Therefore, more and more studies have been conducted on its antioxidant properties. The above series of studies lead to a similar conclusion that bilirubin production is related to antioxidant defense mechanisms and that higher serum bilirubin concentrations are associated with lower hazard ratios for ischemic stroke. In any case, all these findings merely reflect the role of bilirubin in stroke prevention. Little evidence exists about what happens to bilirubin when an ischemic stroke occurs.

Therefore, it is necessary to clarify the role of serum bilirubin in AIS patients further. In this study, a healthy Control Group is set up. It is found that the TBIL, DBIL, and IBIL of AIS patients are higher than those of the Control Group, and those of male patients are higher than female patients (*P* < 0.05). According to the NIHSS scores at admission, AIS patients are divided into three subgroups: Mild Group, Moderate Group, and Severe Group. It is found that the bilirubin levels increase with the increase in the severity of the disease at admission. Multivariate ordinal Logistic regression analysis shows that only DBIL was associated with the severity of the disease, and the higher the NIHSS scores are, the higher the levels of DBIL are. After further drawing the ROC curve, it is found that DBIL > 2.85 μmol/L has a particular role in predicting severe stroke (AUC was 0.596) and is relatively sensitive (sensitivity was 72.7%).

The effect of DBIL on early functional outcome is investigated according to the mRS scores at 7 ± 2 days. It is found that patients with higher DBIL levels at admission have relatively higher mRS scores at 7 ± 2 days, and there is a certain correlation. The ROC curve shows that its AUC for predicting poor early functional outcome is 0.582, the cut-off value is 2.55 μmol/L, and it also has relatively high sensitivity (80.0%). However, the specificity for predicting either the severity (43.5%) or early functional outcome (36.6%) of AIS patients is relatively low. Then, this study combines the DBLR with LYM count to analyze the value in predicting the severity and poor early functional outcome of AIS patients. Further binary Logistic regression screening shows that DBIL is not an independent factor representing poor early functional outcome, which is basically consistent with the results of the study by Pineda et al. (2008). In these patients with AIS, any significant relationship between TBIL and IBIL at admission and initial stroke severity or discharge outcomes is not found. It is unclear why DBIL, but not TBIL, is associated with initial stroke severity, and this difference requires further investigation. Previous studies also did not distinguish between the different effects of TBIL and DBIL levels on AIS. However, studies on patients with general liver disease suggested that DBIL levels might have higher sensitivity than TBIL levels in the diagnosis of diseases. Therefore, combined with previous studies, this study speculates that DBIL can be used as a predictor of the severity and early functional outcome of stroke. When DBIL > 2.55 μmol/L in patients with AIS, the potential severity and poor early functional outcome of the patient should be considered. In conclusion, DBIL elevation is associated with stroke severity and poor early functional outcome. However, it is not an independent factor for poor early functional outcome. Therefore, DBIL can be used as a simple predictor to guide the treatment of early ischemic stroke, which provides preliminary evidence for clinical exploration.

The possible mechanism by which DBIL is specifically associated with AIS severity and early functional outcome may be related to the selective transport of DBIL across the blood-brain barrier. Unlike TBIL and IBIL, DBIL is water-soluble and may more easily enter the brain parenchyma during acute ischemia, participating in local inflammatory responses or oxidative stress. In the acute phase of AIS, cerebral ischemia-reperfusion injury induces excessive reactive oxygen species production and inflammatory cell infiltration. DBIL may accumulate in ischemic brain tissue, leading to increased neurotoxicity, while its antioxidant effect is insufficient to offset the damage, resulting in more severe neurological deficits and worse early functional outcome. This may explain why DBIL, rather than TBIL or IBIL, shows a significant correlation with AIS outcomes in this study.

Our finding that DBIL, rather than TBIL or IBIL, showed the strongest association with stroke severity is consistent with the observations of Xu et al. (2026),who reported that DBIL demonstrated superior predictive value for adverse outcomes compared with IBIL and TBIL in AIS patients undergoing mechanical thrombectomy (Xu et al., 2026). The consistency across different AIS populations strengthens the credibility of DBIL as a clinically relevant biomarker. Furthermore, Qiu et al. (2025) extended this association to hemorrhagic stroke, showing that elevated TBIL levels predict higher 28-day mortality in patients with intracerebral hemorrhage. Collectively, these studies suggest that the prognostic value of bilirubin may transcend stroke subtypes, although the optimal bilirubin fraction (DBIL vs. TBIL) may vary depending on the specific outcome and population.

### 5.2 Significance of this study

To or knowledge, this is the first study to investigate the value of DBLR in predicting the severity and early in-hospital functional outcome of AIS. Some previous studies found that TBIL was negatively correlated with the severity and early in-hospital functional outcome of AIS, while some found that it was positively correlated. However, after the analysis of this study, it is found that with the increase in the severity of the disease, the bilirubin levels increased. Otherwise, multivariate regression analysis shows that only DBIL is associated with the severity and early in-hospital functional outcome of patients. However, ROC curve analysis finds that DBIL has relatively high sensitivity and low specificity in predicting the severity and early in-hospital functional outcome of patients. Therefore, it may have exploratory value to further explore DBLR as a composite marker of oxidative stress and immune response in AIS.

This study suggests that the DBLR level at admission is closely related to the severity of stroke. ROC curve analysis shows that DBLR has a higher ability to predict severe stroke than DBIL (AUC: 0.612 vs. 0.596), with a best diagnostic value of 2.86, and DBLR had a higher specificity (79.4%) than DBIL. Therefore, the exploratory cut-off value of 2.86 may provide reference for early risk stratification but requires external validation before clinical application. DBLR is also significantly different between the Good early in-hospital functional outcome Group and the Poor early functional outcome Group. Spearman correlation analysis shows that DBLR is positively correlated with poor early functional outcome. Further binary logistic regression analysis showed that continuous DBLR was independently associated with poor short-term in-hospital functional status (OR = 1.234, *P* = 0.001). The exploration cutoff of DBLR > 2.86 (OR = 5.169, *P* = 0.005) showed a similar directional trend, but this threshold was derived from the current single-center cohort and requires external validation before clinical application. The categorical analysis using the ROC-derived cutoff should be interpreted with caution, as the cutoff was identified and tested in the same dataset. with limited predictive performance (AUC < 0.7) (AUC: 0.650, *P* < 0.001, sensitivity: 69.8%, specificity: 58.5%). Therefore, this study suggests that DBLR has weak-to-moderate exploratory value in assessing stroke severity and early in-hospital functional status.

Notably, in the multivariate ordinal logistic regression analysis (Table 5), DBLR yielded an OR < 1, which seems inconsistent with three independent lines of evidence: (1) DBLR increased progressively across mild (1.99), moderate (3.61), and severe (3.75) stroke subgroups (*P* < 0.001); (2) DBLR was positively correlated with admission NIHSS scores (*r* = 0.321, *P* < 0.001); and (3) DBLR > 2.86 was an independent predictor of poor short-term in-hospital functional status in binary logistic regression (OR = 5.169, *P* = 0.005).

This discrepancy is a well-documented statistical suppression effect caused by the simultaneous inclusion of three mathematically interdependent variables: DBIL, lymphocyte count (LYM), and DBLR (calculated as DBIL/LYM). When DBIL is held constant in the model, a higher DBLR necessarily corresponds to a lower LYM count—and lower LYM is itself independently associated with worse stroke outcomes (*r* = −0.274, *P* < 0.001). This mathematical coupling distorts the regression coefficient sign for DBLR in this specific model configuration.

Critically, this statistical artifact does not undermine the clinical utility of DBLR. Multiple analyses confirmed that DBLR may provide modest additional information when combined with NIHSS beyond DBIL or LYM alone: it achieved higher AUC and specificity than DBIL in both severity and short-term in-hospital functional status ROC analyses, and only DBLR (not DBIL or LYM) remained an independent predictor of poor short-term outcomes after full adjustment for confounders.

### 5.3 Limitations

First, this study used mRS at 7±2 days as the primary early functional outcome, whereas 3-month mRS is the gold standard for long-term functional recovery. Our findings are therefore limited to short-term in-hospital functional status and cannot be extrapolated to long-term outcomes. Nevertheless, we provide context for this choice: (a) The average length of hospital stay for AIS patients in China is 7–10 days, and the 7 ± 2-day time point aligns with routine discharge assessment, ensuring complete data collection with < 1% loss to follow-up during the acute phase; (b) Neurological deficits generally stabilize by 7–10 days after stroke onset, and for the 20–30% of patients who die during hospitalization or develop permanent severe disability, their long-term outcomes are effectively determined at this early stage; (c) This study was specifically designed to investigate admission biomarkers for early risk stratification and in-hospital management, whereas 3-month outcomes are strongly influenced by post-discharge factors (e.g., rehabilitation access, home care quality, and late complications) that are unrelated to admission biomarker levels. To address this limitation, we have initiated a prospective 3-month follow-up study of this entire cohort and will report those findings separately. Second, this is a single-center retrospective study without randomization. Although we adjusted for known confounders, residual confounding cannot be entirely excluded. Third, the sample size is relatively modest; larger multicenter prospective studies are needed to validate our findings. Fourth, only admission biomarker levels were analyzed, without dynamic monitoring of changes over the acute phase. Fifth, the AUC values of DBIL and DBLR are all below 0.7, indicating limited independent predictive ability. These biomarkers should therefore only serve as auxiliary tools to complement clinical assessment, not as standalone predictors. Their clinical value should not be overinterpreted. Future studies should include larger multicenter cohorts, longer-term follow-up, dynamic biomarker monitoring, and mechanistic experiments to further validate and extend our findings.

## 6 Conclusion

DBIL and DBLR are simple, low-cost, and rapidly available routine blood biomarkers that can serve as auxiliary indicators for early severity assessment and short-term in-hospital functional status of acute ischemic stroke. They are particularly valuable in resource-limited settings where advanced neuroimaging or frequent neurological assessments are not readily available. DBLR > 2.86 as a continuous variable was independently associated with poor early functional outcome. However, due to their limited independent predictive power (all AUCs < 0.7), these biomarkers must always be used in combination with clinical indicators such as admission NIHSS scores. Future prospective studies with 3-month follow-up are needed to evaluate their role in predicting long-term functional recovery.

## Data Availability

The raw data supporting the conclusions of this article will be made available by the authors, without undue reservation.
