# Supplementary material for: Admission direct bilirubin and direct bilirubin-to-lymphocyte ratio as exploratory markers of early severity and in-hospital functional status in acute ischemic stroke
Source: Front Stroke. 2026 Aug 7;5:1800285. doi: 10.3389/fstro.2026.1800285 (PMC13493299; doi:10.3389/fstro.2026.1800285)
Supplement: Supplementary file 1 [file Table_1.docx]

**Table S1.** Separate ordinal logistic regression models for DBIL, LYM, and DBLR predicting severity (AIS Group)

| *Model* | *Predictor* | *OR (95% CI)* | *P value* |
| --- | --- | --- | --- |
| Model 1 | DBIL (per 1 μmol/L increase) | 1.581 (1.223–2.043) | **<0.001** |
| Model 2 | LYM count (per 1×10⁹/L decrease) | 0.724 (0.602–0.871) | **0.001** |
| Model 3 | DBLR (per 1-unit increase) | 1.326 (1.087–1.618) | **0.006** |
