# Supplementary material for: Admission direct bilirubin and direct bilirubin-to-lymphocyte ratio as exploratory markers of early severity and in-hospital functional status in acute ischemic stroke
Source: Front Stroke. 2026 Aug 7;5:1800285. doi: 10.3389/fstro.2026.1800285 (PMC13493299; doi:10.3389/fstro.2026.1800285)
Supplement: Supplementary file 2 [file Table_2.docx]

**Table S2** Pairwise comparison of ROC curves (DeLong test)

| *Comparison* | *ΔAUC* | *SE* | *95%CI* | *Z值* | *P value* |
| --- | --- | --- | --- | --- | --- |
| NIHSS+DBLR vs NIHSS | 0.002 | 0.00297 | -0.00337-0.00827 | 0.826 | 0.408 |
| DBLR vs NIHSS | 0.101 | 0.0433 | 0.0157-0.185 | 2.323 | 0.020 |
| DBLR vs NIHSS+DBLR | 0.103 | 0.0419 | 0.0209-0.185 | 2.459 | 0.014 |
| DBLR vs DBIL | 0.033 | 0.0310 | -0.0283-0.0931 | 1.046 | 0.296 |
